# The New Poor-Law Orders: The Duties, and a Policy for Medical Officers

**Published:** 1914-01-17

**Authors:** 


### THE NEW POOR-LAW ORDERS.

### The Duties, and a Policy for Medical Officers.

The new Poor-Law Orders, the main points of which were summarised in our last issue, are in so many ways an improvement upon the old Orders they displace that it will be generally felt as a matter for regret that more drastic reforms have not been introduced in the case of the sick inmates in workhouses. It may be argued that the new Orders were meant to be mainly a consolidation of the old Orders, and that radical changes were therefore debarred. The changes that have been made are sufficiently important, however, to make one suspect that the reason the problem of the sick has not been dealt with more efficiently lies in the absence from the Departmental Committee which framed the Draft Orders of any member with an expert knowledge of the needs of the sick and of the administrative difficulties with which the management of the sick wards in mixed workhouses is beset.

Nevertheless, two very important reforms have been introduced—reforms which cannot but have a very great effect in improving the position of the sick inmates of many workhouses. The first reform is the duty laid on all workhouse medical officers of keeping a full record of their examinations of their patients, the treatment adopted, and the progress of each case. The second reform is the insistence upon the provision of fully trained nurses in every workhouse and the greater control over the sick wards given to the superintendent nurse or head nurse, as the case may be, acting under the medical officer. The effect of these reforms in many workhouses, especially the smaller ones in country districts, will be little short of revolutionary. The Royal Commission on the Poor Laws (1905) was unanimous in deploring the inadequacy of the medical arrangements made in many workhouses by Boards of Guardians. It was proved in the evidence given before that Commission that in not a few Unions the workhouse medical officer found himself able to attend only irregularly and infrequently, and to give only a perfunctory service.

### Additional Work of the New Records.

One inspector stated that "some medical officers frequently stay for a few minutes only in the house, and very rarely long enough to make an examination of the bodily condition of the patients and of their surroundings." One workhouse medical officer frankly admitted that he was not able to give the patients as much attention as they ought to have, and he blamed the Guardians, who would neither remunerate him sufficiently nor provide him with an assistant. If the requirements of the new Orders are carried out scamped work of this kind will no longer be possible. In those workhouses where the medical work has been neglected in the past the medical officers will find that the time they must devote to their duties in the house will be enormously increased. Indeed, even in those workhouses where the work has been conscientiously performed—and to the credit of the medical profession one is glad to find the Commission admitting that such was the case in the majority of workhouses as the result of great self-sacrifice on the part of the medical officers—even in these cases the keeping of the new records required will necessitate many additional hours of work.

### Medical Salaries and How to Raise Them.

Now, in discussing the unsatisfactory character of the medical work in many workhouses the Royal Commission were unanimous in laying the blame for it upon the inadequate salaries offered to medical officers, and in addition in many cases to the system of requiring medical officers to provide at their own expense all the medicines they prescribed. It is, therefore, disappointing to find that neither in the Order nor in the circular-letter accompanying it is anything said about any increase in the medical officer's remuneration, nor about the provision of professional or clerical assistance for him, and that the Guardians are not prohibited from entering into a contract with him to supply medicines and medical appliances at his own expense. Medical officers of workhouses are, in fact, face to face with this position. Their work, which a Royal Commission has stated in the strongest terms to be inadequately and miserably remunerated, is to be very considerably increased, and they are to be left individually to fight their respective Boards of Guardians in order to obtain fair remuneration and adequate assistance. The necessity for co-operation in the face of such a position becomes urgent. It should lead at once to every workhouse medical officer joining the Poor-Law Medical Officers' Association and to the formulation by that body of a minimum scale of remuneration graduated according to the number of beds in the sick wards of each workhouse. In the struggle that is bound to ensue before proper conditions can be obtained, the workhouse medical officers have the right to expect, and will, no doubt, receive full support from the rest of the profession. With the increase in the control of the medical profession which the Insurance Act has brought about, it is to the interest of every member of the profession to see that the claims of institutional medical men for adequate remuneration are properly met, for the salaries they receive are likely to be used in the future as a criterion of the remuneration to be paid by the State for other medical services.

### Medical Officer and Workhouse Master.

As important to the medical officer as his remuneration and the work required of him is his position as regards the control of the sick wards and of the nursing staff. In this respect also the new Orders are far from satisfactory, but they do improve to a certain extent upon the existing conditions in many workhouses. In all workhouses, in which the Local Government Board requires or sanctions the appointment of a superintendent nurse, the matron of the workhouse will be excluded from the sick wards, and the duties she would otherwise perform in respect to those wards are to be carried out by the superintendent nurse acting under the directions of the medical officer. This change will lead undoubtedly to a better administration of the sick wards, and removes a condition of things which has given rise to much friction and unpleasantness in the past. The position of the medical officer is also improved by the numerous instances in which the Guardians are specifically ordered to obtain his written recommendations before making regulations. His position as regards the workhouse master is not so satisfactory. In the old Orders the duties of the medical officer preceded those of the master, and the duties of the latter were so worded that the relative position of the two officers was not clearly defined, but it was always considered that they were independent the one of the other, except in so far as it was the duty of the master "to enforce the observance of all regulations for the government of the workhouse," and to carry out the instructions of the medical officer in certain specified matters. In the new Orders, however, the duties of the master are given precedence over those of the medical officer, and it is definitely stated that "the master shall govern and control, subject to the directions of the Guardians, the institution and the officers thereof."

### An Impossible Relation.

When it is remembered how often in the past the medical officer has had to champion the cause of the inmates, and especially of the sick inmates, against the master, it is obvious that this subordination of the medical officer to the master will have to be resisted strenuously until the administration of the sick wards is separated entirely from that of the rest of the workhouse and placed under the supreme control of the medical officer. Another regulation which makes the position of the medical officer worse than it was under the old Orders is that which requires all officers to produce any documents kept by them or in their custody whenever required by the clerk to the Guardians. In the case of documents referring to the medical history of inmates, this power should never have been given to any individual officer. Such documents should only be disclosed to the Board of Guardians or a statutory committee of Guardians. So long as this clause remains in force it will behove medical officers to bear it in mind in deciding what documents to preserve, and what facts to record on documents liable to be disclosed under it. The increased powers given to Boards of Guardians as regards classification in, and the general administration of, the workhouse is likely to lead to great variations in the routine in different institutions. In many respects such variations from one stereotyped routine, often ill-adapted to local conditions, are to be welcomed, but if abuses are to be prevented the inspection of workhouses will have to be more stringent and more frequently made than is the case at present.

### A Pregnant New Clause.

It is interesting to note, and it may prove in the future a matter of very great importance, that the new Orders contain a clause which was not present in the Draft Order—a clause directing the Guardians to comply with any directions from time to time given by the Local Government Board in regard to the classification of the inmates. Under this clause it would be possible for the Local Government Board to initiate changes which would have very far-reaching effects.

### Where the Orders Need Improvement.

Space will permit of only a brief reference to the following points in which the new Orders might have been improved:—

1. It is much to be regretted that no attempt has been made to compel Boards of Guardians to employ a minimum medical and nursing staff. It would be impossible, no doubt, to fix for all workhouses definite minimal numbers which would be of much value, but it would have been easy to frame a clause making it compulsory upon each Board of Guardians to provide a staff in accordance with a scheme to be drawn up by the local Poor-Law inspector after consultation with the medical officer of the workhouse and the Local Government Board.

2. The master is given the power in cases of urgency to place an inmate, when the medical officer is unable to examine him on his admission, in a ward appropriate for his cure and treatment. Such powers in the hands of a layman may lead to serious mistakes being made. In such cases the inmate should be treated in the receiving ward, pending the arrival of the medical officer.

3. No provision is made for the medical officer's half-yearly reports on the condition of the workhouse to be forwarded to the Local Government Board. It is obvious that the Local Government Board ought to be cognisant of the contents of these reports, and ought where necessary to bring pressure upon the Guardians to carry out the recommendations contained in them.

